# Design of three-component essential oil extract mixture from *Cymbopogon flexuosus*, *Carum carvi,* and *Acorus calamus* with enhanced antioxidant activity

**DOI:** 10.1038/s41598-024-59708-x

**Published:** 2024-04-22

**Authors:** Hamza Assaggaf, Mohamed Jeddi, Hanae Naceiri Mrabti, Amine Ez-zoubi, Ahmed Qasem, Ammar Attar, Bey Hing Goh, Sang Loon Tan, Abdelhakim Bouyahya, Khang Wen Goh, Naoufal El Hachlafi

**Affiliations:** 1 Department of Laboratory Medicine, Faculty of Applied Medical Sciences, Umm Al-Qura University, 21955 Makkah, Saudi Arabia; 2 Laboratory of Microbial Biotechnology and Bioactive Molecules, Faculty of Sciences and Technologies, Sidi Mohamed Ben Abdellah University, Imouzzer Road, P.O. Box 2202, Fez, Morocco; 3 Laboratory of Applied Organic Chemistry, Faculty of Sciences and Techniques, Sidi Mohamed Ben Abdellah University, Route d’Imouzzer, Fez, Morocco; 4 Sunway Biofunctional Molecules Discovery Centre, School of Medical and Life Sciences, Sunway University, 47500 Bandar Sunway, Selangor Malaysia; 5 Laboratory of Human Pathologies Biology, Faculty of Sciences, Mohammed V University in Rabat, BP 1014, Rabat, Morocco; 6 Faculty of Data Science and Information Technology, INTI International University, Nilai, Malaysia; 7 Faculty of Health, Australian Research Centre in Complementary and Integrative Medicine, University of Technology Sydney, Ultimo, Australia; 8 Euromed Research Center, Euromed Faculty of Pharmacy and School of Engineering and Biotechnology, Euromed University of Fes (UEMF), Meknes Road, 30000 Fez, Morocco

**Keywords:** Essential oils, Experimental mixture design, Desirability function, DPPH, ABTS, Antioxidant, Food preservation, Drug discovery, Chemistry

## Abstract

The development of novel antioxidant compounds with high efficacy and low toxicity is of utmost importance in the medicine and food industries. Moreover, with increasing concerns about the safety of synthetic components, scientists are beginning to search for natural sources of antioxidants, especially essential oils (EOs). The combination of EOs may produce a higher scavenging profile than a single oil due to better chemical diversity in the mixture. Therefore, this exploratory study aims to assess the antioxidant activity of three EOs extracted from *Cymbopogon flexuosus*, *Carum carvi*, and *Acorus calamus* in individual and combined forms using the augmented-simplex design methodology. The in vitro antioxidant assays were performed using DPPH and ABTS radical scavenging approaches. The results of the Chromatography Gas-Mass spectrometry (CG-MS) characterization showed that citral (29.62%) and niral (27.32%) are the main components for *C. flexuosus*, while d-carvone (62.09%) and d-limonene (29.58%) are the most dominant substances in *C. carvi*. By contrast, β-asarone (69.11%) was identified as the principal component of *A. calamus* (30.2%). The individual EO exhibits variable scavenging activities against ABTS and DPPH radicals. These effects were enhanced through the mixture of the three EOs. The optimal antioxidant formulation consisted of 20% *C. flexuosus*, 53% *C. carvi*, and 27% *A. calamus* for DPPHIC50. Whereas 17% *C. flexuosus*, 43% *C. carvi*, and 40% *A. calamus* is the best combination leading to the highest scavenging activity against ABTS radical. These findings suggest a new research avenue for EOs combinations to be developed as novel natural formulations useful in food and biopharmaceutical products.

## Introduction

In the last two decades, special interest has been dedicated to exploring novel, effective, and safe antioxidants from natural products to avoid oxidative damage of living cells induced by reactive chemical species (RCS). Free radicals represent the major RCS implicated in the oxidation process. They contain an unpaired electron on the valence orbital and are highly reactive^1,2^. Free radicals such as hydroxyl radical (HO·), hydrogen peroxide (H_2_O_2_), superoxide anion (O_2_·^−^), oxygen singlet (^1^O_2_), peroxynitrite (NO_3_^−^) and nitric oxide (·NO) radical may cause the alteration of vital macromolecules, including lipids, DNA, and proteins^3,4^. This event leads to cell injury and disruption of normal homeostasis^5^, and thus trigger a number of pathological disorders, such as cancer and inflammatory diseases^6,7^. Therefore, the use of external sources of antioxidants can help maintain a certain equilibrium between free radicals and the antioxidant system and thus preserve physiological functions. However, the available commercialized antioxidants such as butylated hydroxyanisole (BHA) and butylated hydroxytoluene (BHT) have recently been demonstrated to be hazardous for human health^1,8,9^. In fact, researchers have recently turned their attention to new preservatives, such as the development of new and non-toxic antioxidant formulations from natural resources, especially essential oils (EOs). EOs are defined as multifaceted bioactive components that comprise various classes of chemical compounds, including monoterpenes, alcohols, sesquiterpenes, aldehydes, phenols, esters, ethers, ketones^10–13^. They possess important antioxidant properties and are found applications as natural antioxidants in the food industry and pharmacy^14–16^.

*Cymbopogon flexuosus* is commonly known as lemongrass and belongs to the Poaceae family, which is also known as the grass family. *C. flexuosus* has shown several traditional uses and is known for its therapeutic properties, including antimicrobial, anti-inflammatory and anticancer propertie^17^. *C. flexuosus* is widely used in cooking to add a citrusy and aromatic flavor to dishes. It is often used in soups, curries, marinades, and teas^18^.

*Carum carvi*, commonly known as caraway and belonging to the Apiaceae family, is present in natural vegetation and is also deliberately grown as both a medicinal herb and a culinary plant in various regions around the world^19^. *C. carvi* has been traditionally used for various medicinal purposes. It has shown carminative properties, helping to alleviate digestive problems such as indigestion, bloating, and flatulence. *C. carvi* has also been used to relieve spasms and cramps in the digestive system and could contribute to treating inflammation and respiratory conditions^20^.

*Acorus calamus*, commonly known as sweet flag or calamus, is a perennial herbaceous plant that has been utilized for various medicinal and common purposes^21^. Belonging to the Acoraceae family, this herbaceous plant has a long history of traditional use in different cultures around the world. Calamus has a range of potential medicinal uses. Traditionally, it has been used as a remedy for digestive ailments. The rhizomes of *A. calamus* are believed to possess carminative properties, aiding in the relief of indigestion, bloating, and gas^22^. Moreover, it has been used as an expectorant, helping to ease respiratory congestion and coughs. Some traditional medicinal systems also suggest its use for mental clarity and memory enhancement. Besides its traditional applications, *A. calamus* has been employed in various other common uses. One notable use is as a fragrant herb in the perfume and cosmetics industries. The aromatic qualities of the plant make it a popular choice for adding fragrance to products^23^.

Interestingly, recent reports have demonstrated that the use of combined EOs can enhance their antioxidant effect^24–26^. While the synergistic interactions between EOs are yet fully understood, such knowledge, particularly regarding the proportions and interactions mechanisms between each component in a mixture, is important in order to discover novel and effective combinations. With this in mind, we designed a new conception of the mixture to assess the antioxidant ability generated through the interactions of three oils from *Cymbopogon flexuosus, Carum carvi, Acorus calamus* using an augmented-simplex design methodology. This method provides the necessary EOs concentrations that exhibit synergistic antioxidant effect.

## Materiel and methods

### Plant material and extraction of EOs

The plants *Acorus calamus* L. (aerial parts), *Cymbopogon flexuosus* L. (aerial parts), and *Carum carvi* L. (seeds) were harvested from a farmer (Mernissa) in the Taounate region (34° 39′ 03″ N, 4° 16′ 40″ W), North of Morocco. The plants identification was performed at the Scientific Institute, Mohammed V University in Rabat, by Pr. Mohamed Ibn Tattou under voucher codes RAB 11,415–11,417. The current study conformed to all applicable institutional, national, and international guidelines and regulations. The samples were dried in appropriate conditions (continuous ventilation at dark place) and then extraction of EOs was undertaken. In fact, an amount of 100 g of dried plants was subject to hydro-distillation for 180 min using Clevenger-type device. The distilled oils were recuperated and dehydrated by anhydrous sodium sulfate, filtered, and kept at 4 °C, pending upcoming tests.

### GC–MS analysis

The chemical characterization of the three EOs was performed using chromatography (GC) (Trace GC-Ultra) attached with mass spectrometry (MS) (Quadrapole, Polaris Q) (GC–MS) as described by Benkhaira et al.^27^. The machine is equipped with a non-polar HP-5MS capillary column (30 m, 0.32 mm × 0.25 µm). The temperature of the injector and detector was set at 280 and 300 °C, respectively. The column temperature was monitored at 50 °C for 5 min and then at 180 °C for 4 °C/min and. Helium (He) was used as the carrier gas (1.3 mL/min). A volume of 0.5 µL of EOs was manually injected. The chemical identification of samples components was established based on the comparison of their respective retention index (RI) (obtained and calculated based on homologous series of alkanes ranging from C_8_ to C_24_) and mass spectral (MS) fragmentation patterns with those recorded in the published articles^28,29^. Moreover, the individual constituents were quantified through internal normalization of the total area of peaks noticed in each chromatogram. The MS of each constituent was completed according to the data stored in chemical libraries (NIST/EPA/NIH MASS SPECTRAL LIBRARY Version 2.0, July 1, 2002) via computer matching. To determine the relative peak area percentage of a mixture, such as an essential oil (EO), the initial step involves the summation of all individual peak areas. Then, to calculate the percentage of each EO component, we divide its individual area by the total area and multiply the result by 100.

### Antioxidant assays

#### DPPH radical scavenging assay

2,2-diphenyl 1-picrylhydrazyle (DPPH) was used to determine the antiradical activity of three EOs as well as their different combinations generated by experimental design approach. A modified version of protocol by El Hachlafi et al.^30^ was adopted for this subject. Specifically, 200 mL aliquots samples of different concentrations previously prepared in methanol were added to 1.4 mL of DPPH solution (0.004%). The obtained solutions were then incubated for 35 min at dark place. Afterwards, the optical density was established at 517 nm using a spectrophotometer. Butylated hydroxytoluene (BHT) was used as a reference.

The scavenging ability of the EOs was estimated using Eq. (1):

1 $$DPPH scavenging ability \left(\%\right)= \frac{({OD}_{c}-{OD}_{EO})}{{OD}_{c}}\times 100$$

where OD_C_ and OD_EO_ is the optical density of the control and tested EOs, respectively.

#### ABTS radical scavenging activity

2,2′-azino-bis 3-ethylbenzothiazoline-6-sulphonic acid (ABTS) radical scavenging method was performed as indicated by Al-Mijalli et al.^10^, with minor modifications. Briefly, the cationic ABTS radical was produced by reacting 10 mL of the solution of ABTS (2 mM) with 0.1 mL of potassium persulfate solution (70 mM). The obtained combination was kept in a dark place for 14 h. Then, the mixture was diluted with methanol until an optical density of 0.700 ± 0.02 at 734 nm was obtained. A volume of 0.2 mL of samples at different concentrations was mixed with 2 mL of the diluted ABTS solution. After incubation for 2 min, the optical density was determined against a blank (methanol) at 734 nm. ABTS scavenging potential was represented as IC_50_ (μg/mL) ± SD (*n* = 3). Butylated hydroxytoluene (BHT) was used as a reference drug.

### Experimental design

#### Mixture design

An augmented simplex-centroid design technique was adopted to identify the optimum antioxidant effect of the combination of EOs of *C. flexuosus*, *C. carvi*, *A. calamus* as described by Benkhaira et al.^27^. The EO system constituents are listed in Table 1. Each EOs may possess a value ranging between 0 and 1 in the mixture, and the sum of three components is equal to 1 (Table 1). In addition, the DPPH_IC50_ and ABTS_IC50_ responses were used to assess the antioxidant ability of the studied EOs.

**Table 1:** Identification of independent variables used in the mixture.

Components	Composition (µg/ml)		
	Coded variables	Level−	Level +
*C. flexuosus*	X_1_	0	1
*C. carvi*	X_2_	0	1
*A. calamus*	X_3_	0	1
Sum of proportions			1

#### Experimental matrix and mathematical model

**Figure 1:**
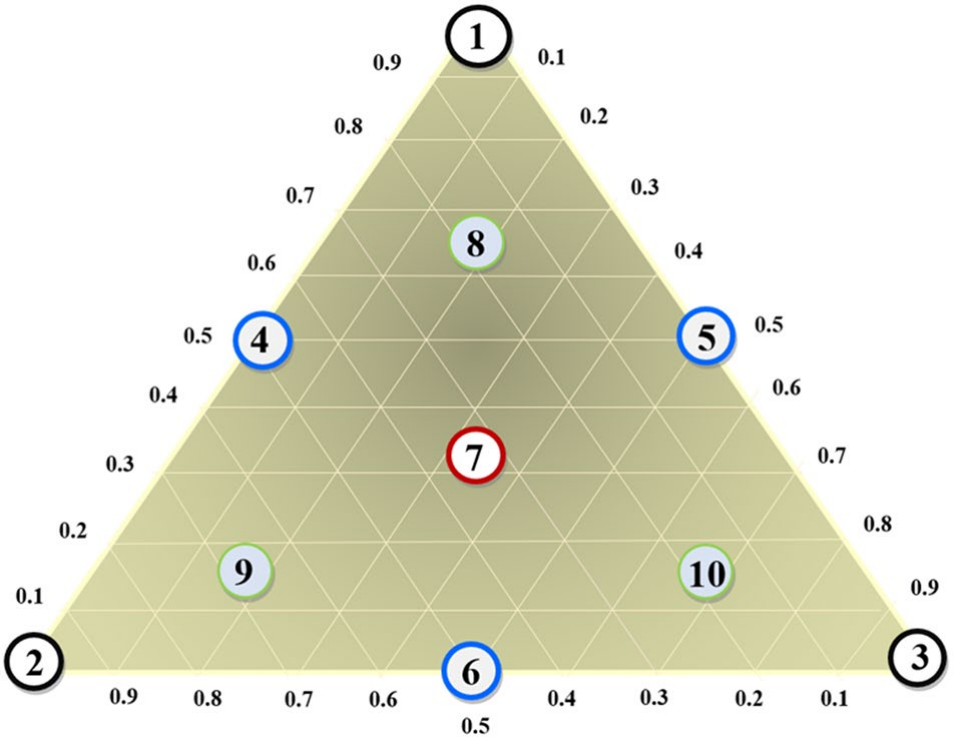
An overview of the simplex-centroid design for three compounds.

In the current investigation, a total of 10 trials were performed and are depicted as an equilateral triangle (Fig. 1), comprising three pure components (1), corresponding–to triangle apexes (X_1_, X_2_, X_3_), binary combination (0.5/0.5) marked by midpoints of the triangle (X_4_, X_5_, X_6_), equal proportional of the three components (0.33/0.33/0.33), detected at the gravity center of the triangle (X_7_). This trial was carried out in three separate replicates, and finally the three control points (X_10_, X_11_, X_12_), comprising ternary mixtures (0.67/0.16/0.16). A cubic model was used to indicate responses according to the independent variable using the formula below (2):

2 $$Y={{\Omega }_{1}{\text{X}}}_{1} + {{\Omega }_{2}{\text{X}}}_{2} + {{\Omega }_{3}{\text{X}}}_{3} + {\Omega }_{12}{{\text{X}}}_{1}{{\text{X}}}_{2} + {\Omega }_{13}{{\text{X}}}_{1}{{\text{X}}}_{3}+ {\Omega }_{23}{{\text{X}}}_{2}{{\text{X}}}_{3}+{\Omega }_{123}{{\text{X}}}_{1}{{\text{X}}}_{2}{{\text{X}}}_{3}+\upvarepsilon$$

where Y is the experimental response established by IC_50_ (µg/mL); Ω_1_, Ω_2_, and Ω_3_ are linear regression coefficients, Ω_12_, Ω_13_ and Ω_23_ are binary regression coefficients, Ω_123_ is the ternary regression coefficient, and ɛ is the regression error term.

### Statistical analysis

The ration F_ratio (MSR/MSr)_ between the mean square regression and mean square residual was used to assess the statistical significance of the mathematical model at a 95% confidence level^31^. A greater F-value justifies the variation of the results around their mean. Furthermore, the ratio of the mean square Lack of fit (MS_LOF_) and mean square pure error (MS_PE_) F_ratioLOF/PE_ were employed to examine if the postulated mathematical model was fitted with the observed results. High F _ratioLOF/PE_ values indicate incorrectness of the model^32^. Besides, the coefficient of determination (*R*^2^) was computed to express the quality of the presumed mathematical models. The t-student test was applied to evaluate the importance of the estimated factors^33^. Moreover, the F-test for ANOVA analysis was conducted to verify the significance of the assumed models. The experimental design approach and the graphical and statistical analysis were performed using Design Expert software version 12 and SAS JMP® version 14. The results were established as the means ± SD (*n* = 3).

### Optimization tools

The contour plot and 3D surface for the isoresponse curves were employed to reveal the compromise areas between studied components^34^. Besides, the desirability tool was applied to promote the precise optimal value with a perfect percentage of compromise. This function ensures the maximal adjustment mathematical model, which ranges between 0 and 1. A value of 0 indicates an undesirable response, while a value of 1 signifies a highly desirable response^35^.

## Results and discussions

### Chemical profile of the three EOs

The chemical profile, molecular formula, percentage, and yield of each three essential oils are displayed in Table 2. The yield of the EO extracts from *A. calamus*, *C. carvi* and *C. flexuosus* arew 0.9, 1.1 and 2.3 (v/w). Each of them contains eighteen, nine and thirty phytoconstituents which account for 98.07, 99.44 and 97.61% of the corresponding plants. *β*-Asarone (69.11%) is the most abundant compound in *A. calamus* EO, followed by oxygenated sesquiterpenes (6.17%), sesquiterpene hydrocarbons (4.31%) and asaraldehyde (1.34%) as the single monoterpene (oxygenated).

**Table 2:** Chemical composition of *A. calamus, C. carvi,* and *C. flexuosus* EOs.

Compound	Molecular formula	RI	Area (%)		
			*A. calamus*	*C. carvi*	*C. flexuosus*
*α-*Tricyclene	C_10_H_16_	926	–	–	0.32
*α-*Pinene	C_10_H_16_	943	–	–	0.50
Camphene	C_10_H_16_	952	–	–	2.83
Isolimonene	C_10_H_16_	983	–	–	0.89
d-Limonene	C_10_H_16_	1031	–	29.58	1.32
*α*-Ocimene	C_10_H_16_	1039	–	–	0.37
*β-*Ocimene	C_10_H_16_	1048	–	–	0.35
4-Nonanone	C_9_H_18_O	1030	–	–	2.22
Linalool	C_10_H_18_O	1082	–		2.58
Limonene oxide	C_10_H_16_O	1131	–	0.94	–
Isoneral	C_10_H_16_O	1165	–		0.99
Endo-Borneol	C_10_H_18_O	1172	–	-	0.29
Estragole	C_10_H_12_O	1196	–	0.37	–
p-menth-8-en-2-one	C_10_H_16_O	1208	–	3.01	–
cis-Carveol	C_10_H_16_O	1225	–	1.28	–
Neral	C_10_H_16_O	1238	–	-	27.36
Geraniol	C_10_H_18_O	1239	–	–	4.06
Citral	C_10_H_16_O	1240	–	–	**29.62**
d-Carvone	C_10_H_14_O	1242	–	**62.09**	-
Thiogeraniol	C_10_H_18_S	1255	–	–	3.21
Neryl formate	C_11_H_18_O_2_	1266	–	–	0.41
Carvone oxide	C_10_H_14_O_2_	1279	–	0.36	–
Limonene-1,2-diol	C_10_H_18_O_2_	1342	–	1.24	–
Neric acid	C_10_H_16_O_2_	1347	–	–	1.00
Eugenol	C_10_H_12_O_2_	1356	–	–	1.12
Piperitenone oxide	C_10_H_14_O_2_	1366	–	0.57	–
Geranyl acetate	C_12_H_20_O_2_	1383	–	–	5.95
*β*-Elemene	C_15_H_24_	1384	0.24	–	0.55
Cis-α-bergamotene	C_15_H_24_	1415	–	–	0.34
Caryophyllene	C_15_H_24_	1421	–	–	2.84
trans-Chrysanthemal	C_10_H_16_O	1451	–	–	0.43
Aristolene	C_15_H_24_	1472	0.24	–	–
*γ*-Muurolene	C_15_H_24_	1474	–	–	4.38
*β*-Ionone	C_13_H_20_O	1485	–	–	0.31
Viridifloren	C_15_H_26_O	1493	0.29	–	–
*α*-Muurolene	C_15_H_24_	1499	0.21	–	–
Germacrene D	C_15_H_24_	1500	–	–	0.24
(Z)-Methyl isoeugenol	C_11_H_14_O_2_	1501	2.16	–	–
Isoshyobunone	C_15_H_24_O	1518	1.24	–	–
*β*-Cadinene	C_15_H_24_	1519	0.61	–	–
Kessane	C_15_H_26_O	1536	0.24	–	–
*α-*Cadinene	C_15_H_24_	1538	–	–	0.32
*γ-*Bisabolene	C_15_H_24_	1541	–	–	0.93
*β*-Calacorene	C_15_H_20_	1563	3.01	–	–
Geraniol butyrate	C_14_H_24_O_2_	1565	–	–	0.69
*γ-*Asarone	C_12_H_16_O_3_	1578	3.22	–	–
Caryophyllene oxide	C_15_H_24_O	1581	–	–	1.19
*β*-Asarone	C_12_H_16_O_3_	1583	**69.11**	–	–
Asaraldehyde	C_10_H_12_O_4_	1620	1.54	–	–
tau-Muurolol	C_15_H_26_O	1640	0.44	–	–
Dehydroxy-isocalamendiol	C_15_H_24_O	1645	1.74	–	–
*α*-Asarone	C_12_H_16_O_3_	1703	11.02	–	–
Isocalamenediol	C_15_H_26_O_2_	1760	2.06	–	–
Tatarinoid B	C_12_H_16_O	1847	0.54	–	–
Callitrisin	C_15_H_20_O_2_	1957	0.16	-	-
Monoterpene hydrocarbons			–	29.58	6.58
Oxygenated monoterpenes			1.54	69.86	67.45
Sesquiterpene hydrocarbons			4.31	–	9.6
Oxygenated Sesquiterpenes			6.17	–	1.19
Other			–	–	12.79
Total identified (%)			**98.07**	**99.44**	**97.61**
**Yield (v/w)**			**0.9**	**1.1**	**2.3**

Significant values are in [bold].

The chemical profile of *A. calamus* EO is consistent with other earlier studies conducted in the Polish region, where Szczeblewski et al.^36^ found that asarones were among the most prevalent components. Similarly, Loying et al.^15^ revealed that *β-*asarone is the major compound (82.42%) along with other minor compounds such as calaraene (2.41%) and euasarone (1.92%) in the North-East India region. Generally, *β*-asarone was found in high amount in the East Asia, following by the European areas^37^.

Of the nine compounds detected in *C. carvi*, about 69.86% of the constituents comprise oxygenated monoterpenes, including d-carvone (62.09%) along with other minor compounds such as *p*-menth-8-en-2-one (3.01%), cis-carveol (1.28%) and limonene-1,2-diol (1.24%), as well as d-limonene (29.58%). These results are consistent with another study by Ghannay et al.^38^, in which carvone (58.2%) and limonene (38.5%) are the main detected compounds. Similarly, Lasram et al.^39^ reported that *C. carvi* EOs contained 15 different oxygenated and hydrocarbons monoterpenes which are dominated by carvone (78.85%) and limonene (18.62%).

As for *C. flexuosus*, a total of thirty compounds have been detected. This extract is predominantly composed of oxygenated monoterpenes (67.45%), followed by citral (29.62%) niral (27.32%), and sesquiterpene hydrocarbons (9.6%), whereas minor constituent was detected for monoterpene hydrocarbons (6.58%). The result is consistent with a previous studies where citral and niral were reported to be the major components with a composition of 29.4% and 30.4% respectively^40^. In an investigation of the effect of altitude on chemical profile by Pathania et al.^41^, citral was found as the main compound with a composition ranging from 13.61% to16.14%. Apart from altitude, it was reported that the presence and quantity of chemical constituents may vary due to the intervention of various characteristics, such as climate (temperature, humidity), soil (pH, N:P ratio, mineral) and plant growth cycle^35^.

This reinforces the assertion proposing that ecological, climatic, and nutritional conditions quantitatively and qualitatively affect EO components in plants. Several studies have clarified the positive connection between variations in external and internal factors of plants, including climate, seasonal variation, soil composition, metabolic pathways, and the chemical constituents of EOs^10,42^.

### Antioxidant activity

**Figure 2:**
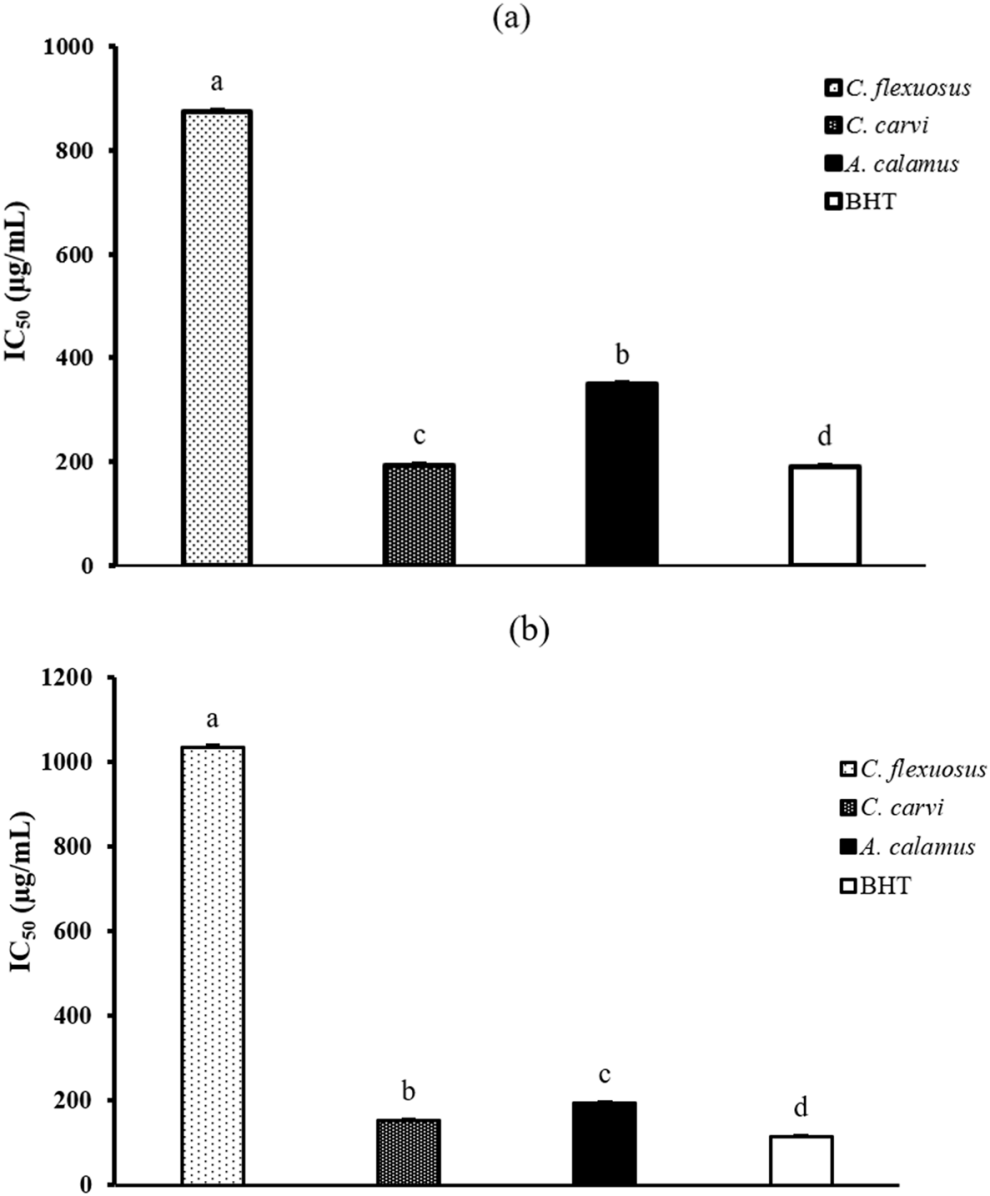
Antioxidant activity of *C. flexuosus*, *C. carvi*, and *A. calamus* EOs examined by (**a**) DPPH, and (**b**) ABTS radicals scavenging assays. Data with the same letter present non-significant variance by ANOVA Tukey’s multiple range test (*p* < 0.05). Results indicated as IC_50_ ± SD of triplicate (*n* = 3).

In the current investigation, two methods were used to assess the antioxidant activity of the EOs extract from *C. flexuosus*, *C. carvi*, and *A. calamus*, namely the DPPH and ABTS radicals scavenging assays. As shown in Fig. 2, the three sources of EOs demonstrated variable antiradical activity on DPPH and ABTS radicals. *C. carvi* possesses high antioxidant activity on both ABTS and DPPH tests with an IC_50_ value of 152.67 ± 3.19 µg/mL and 194.1 ± 2.01 µg/mL, respectively, while *A. calamus* exhibits relatively weaker scavenging activity with an IC_50_ = 193.42 ± 2.52 µg/mL and 349.04 ± 3.73 µg/mL in the corresponding antioxidant assays. The antioxidant activity for *C. flexuosus* is the weakest, with IC_50_ values of 874.22 ± 4.13 µg/mL and 1034.17 ± 5.87 µg/mL for DPPH and ABTS radicals, respectively. The scavenging activity of the three EOs was found to be significantly different (*p* < 0.05) to the standard reference BHT that was used as a positive control (IC_50_ = 114.07 ± 1.42 µg/mL for ABTS and 191.13 ± 0.83 µg/mL for DPPH). The higher antioxidant activity of EOs under optimal conditions results from a combination of factors that support optimal plant growth, metabolism, and defense mechanisms^43^. These conditions create an environment where plants can produce and accumulate higher levels of antioxidant compounds in their essential oils. Additionally, EOs production and antioxidant activity are influenced by nutrient availability. In optimal conditions, plants have access to essential nutrients such as minerals and vitamins, which play a crucial role in the synthesis of antioxidants^1^. Optimal conditions can also influence the expression of genes related to EO constituents and antioxidant synthesis. Under favorable environmental conditions, the plant may upregulate genes implicated in the synthesis of specific antioxidant compounds^44^.

As of to date, there have been several investigations reported on the antioxidant potential of EOs from the three plants using different in vitro tests, including DPPH, ABTS, ORAC, FRAP, and β-carotene bleaching tests^14,15,45–48^.

EOs with the highest antiradical ability may play a pivotal role in diseases prevention, such as neurodegenerative pathologies, cancer, inflammatory disease, and immune system decline^10^. They could protect cell from the harmful effects generated by free radicals, thus preserving proper physiological function^13,49^. It is well known that volatile components possess significant antioxidant activity in different biological systems^11,30^. The antioxidant activity of the studied EOs could be ascribed to the presence of definite chemical classes, including alcohol, ether, ketone, aldehyde, monoterpene, and sesquiterpenes, which demonstrate high ability in neutralizing free radicals and disrupting peroxides^50,51^. In our study, the high scavenging activity of EOs extracted from *C. carvi* could also be due to the abundance of monoterpenes, such as carvone, which possesses a conjugated double bond in its structure and thereby induce antioxidant activities^12,52^. Additionally, carvone has been considered as a protective molecule against lipid peroxidation^12^. Furthermore, the monoterpenes citral, a major component identified in *C. flexuosus* EO, has also shown promising antioxidant properties^53,54^. Similar to monoterpenes, sesquiterpenes can act as direct antioxidants, neutralizing free radicals. Some sesquiterpenes may also modulate cellular signaling pathways related to oxidative stress and inflammation.

### Simplex centroid design

The simplex-centroid design, comprising various mixtures of the three tested EO extracts (*C. flexuosus*, *C. carvi* and *A. calamus*) and the recorded response (DPPH_IC50_ and ABTS_IC50_) of each experiment, is summarized in Table 3. *C. flexuosus, C. carvi* and *A. calamus* essential oils are known for their potential health and wellness benefits. Investigating their combination can contribute to the development of products that relieve oxidative stress and other positive effects. Furthermore, to the best of our knowledge, there is no published work on the combination effect of *C. flexuosus, C. carvi* and *A. calamus* essential oils using experimental design methodology. Indeed, choosing to explore this combination is a commendable and innovative approach.

**Table 3:** Matrix of simplex centroid design and results for DPPH_IC50_, and ABTS_IC50_.

Experiment number^a^	*C. flexuosus*	*C. carvi*	*A. calamus*	Observed responses^b^	
				DPPH _IC50_ (µg/mL)	ABTS _IC50_ (µg/mL)
1	1	0	0	874.22 ± 4.13	1034.17 ± 5.87
2	0	1	0	194.1 ± 2.01	152.67 ± 3.19
3	0	0	1	349.04 ± 3.73	193.42 ± 2.52
4	0.5	0.5	0	361.67 ± 1.02	438.39 ± 0.28
5	0.5	0	0.5	517.82 ± 3.21	410.15 ± 1.14
6	0	0.5	0.5	281.21 ± 1.18	132.06 ± 2.17
7	0.33	0.33	0.33	185.34 ± 1.07	112.27 ± 0.76
8	0.33	0.33	0.33	189.2 ± 0.84	102.33 ± 1.71
9	0.33	0.33	0.33	202.15 ± 0.41	82.25 ± 1.06
10	0.67	0.17	0.17	591.21 ± 1.05	644.13 ± 0.93
11	0.17	0.67	0.17	265.32 ± 1.45	110.48 ± 3.02
12	0.17	0.17	0.67	316.46 ± 1.17	179.02 ± 1.78
BHT				191.13 ± 0.83	114.07 ± 1.42

^a^Experiments were performed after randomization.

^b^The tests were conducted in three independent replicates and established as means ± SD.

The trials (consisting of 12 experiments) were randomized, and each recorded response is the mean of three independent repetitions. The results revealed that the antioxidant activity tests for DPPH_IC50_ and ABTS_IC50_ range from 185.34 ± 1.07 to 874.22 ± 4.13 µg/mL and from 82.25 ± 1.06 to 1034.17 ± 5.87 µg/mL, respectively. Data analysis indicated that the equal-proportional combination of the three studied EO extracts was the best formulations exhibiting the highest scavenging potential on ABTS and DPPH radicals.

#### Statistical validation of postulated model

Variance analysis was conducted to investigate the interaction of various components in the combination, as illustrated in Table 4. The findings demonstrate that the main regression impact was statistically significant for both analyzed responses because the significance of the risk (*p*-value) is lower than 0.05 (0.0024 and 0.0007 for DPPH_IC50_ and ABTS_IC50_, respectively). Evidently, the F_(R/r)_ determined for the two examined responses (19.98 for the DPPH_IC50_ response and 34.29 for the ABTS_IC50_ response) demonstrated that it is greater than the tabular value of F at the 95% confidence level. In addition, the ANOVA F-test demonstrated that both assumed models had no lack of fit, since their *p*-values were less than 0.05 (0.0123 and 0.0319). The calculated F _Ratio (LOF/PE)_ of the studied responses was less than the theoretical value F_LOF/PE_ (F _0.05; 3.2_ = 19.16) at 95% of confidence.

**Table 4:** Variance analysis (ANOVA) for the two adjusted models.

	DPPH _IC50_					ABTS _IC50_				
Model	Freedom degree	Sum of Squares	Mean Square	F _Ratio_	*p-*value	Freedom degree	Sum of Squares	Mean Squares	F _Ratio_	*p*-value
Regression	6	453,343	75,557.20	19.980	0.0024*	6	900,387	150,064	34.2975	0.0007*
Residuals	5	18,908.22	3781.60			5	21,876.92	4375		
Lack of fit	3	18,753.15	6251.05	18.775	0.0123*	3	21,409.179	7136.39	16.514	0.0319*
Pure error	2	155.059	77.53			2	467.737	233.87		
Total	11	472,251.22				11	922,263.91			
*R*^*2*^	0.97					0.98				
*R*^*2*^_adj_	0.88					0.95				
*R*^*2*^_pred_	− 19.00					0.46				

*statistically significant, *R*^*2*^ Coefficient of determination, *adj* Adjusted, *pred* Predicted.

**Figure 3:**
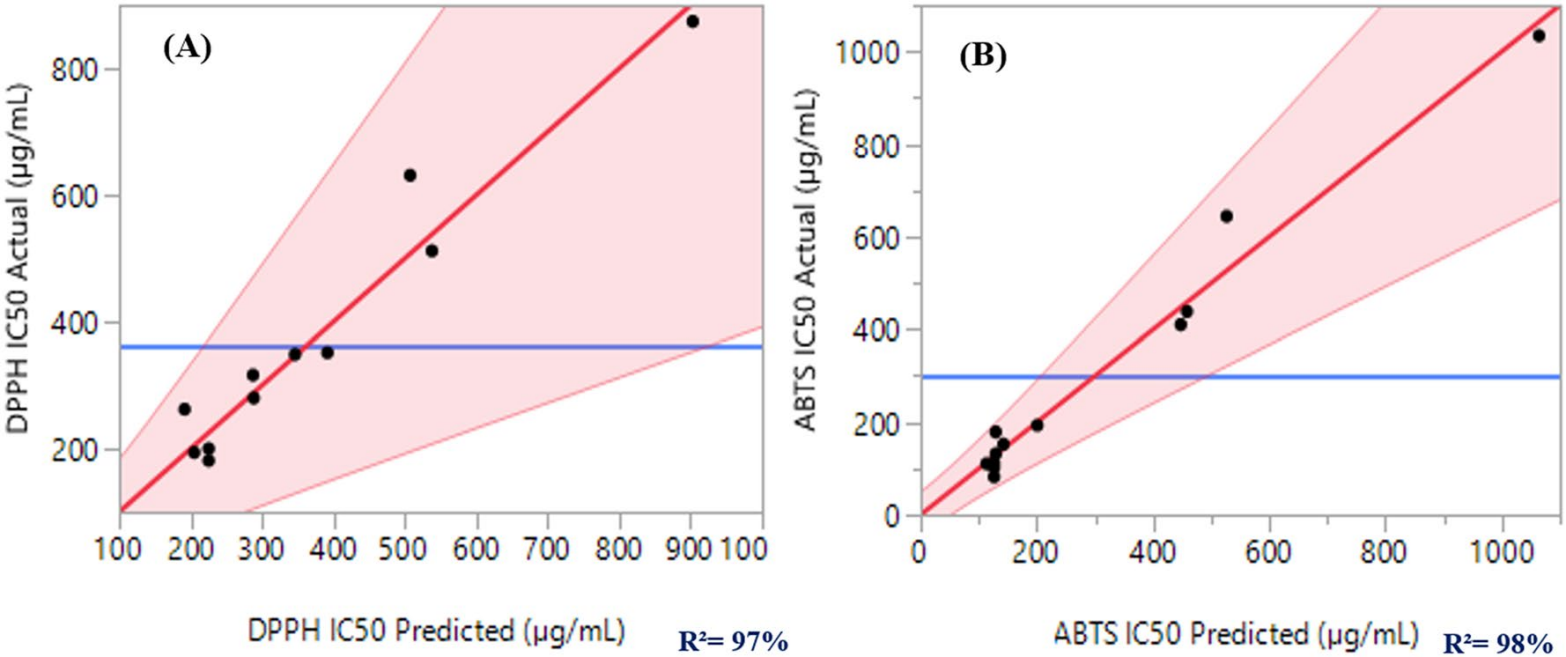
The red lines depict the curve of the experimented values in terms of the expected values for the both responses DPPH_IC50_, and ABTS_IC50._ The blue lines illustrate the actual mean values for the two examined responses.

The importance of a model increases with the coefficient of determination (*R*^2^) and the adjusted *R*^*2*^ values. As a result, according to Table 4, the coefficients of determination (*R*^2^) were 0.97 and 0.98 for DPPH_IC50_ and ABTS_IC50_, respectively, whereas the *R*^*2*^_adj_ was equal to 0.88 and 0.95 for DPPH_IC50_ and ABTS_IC50_, respectively. These analyses reflect a good concordance between the expected and actual data of the assumed models. As a confirmation of these results, the graph (Fig. 3) illustrates a linear curve for the observed values as a function of the expected ones for both studied responses.

#### Compounds effects and adjusted models

Table 5 provides an overview of the special model's estimated regression coefficients. The regression equations with significant coefficients (*p*-values < 0.05) were employed to identify the correlations between all tested factors and the attained responses for DPPH_IC50_ and ABTS_IC50_.

**Table 5:** Coefficients of the two presumed models and their level of significance (*p*-value).

Term	Coefficient	DPPH_IC50_			ABTS_IC50_		
		Estimation	t-Ratio	*P*-value	Estimation	t-Ratio	*P*-value
*C. flexuosus*	Ω_1_	893.10	15.04	< 0.0001*	1064.19	16.66	< 0.0001*
*C. carvi*	Ω_2_	207.53	3.49	0.0174*	142.57	2.23	0.0760
*A. calamus*	Ω_3_	348.14	5.86	0.0020*	200.88	3.14	0.0255*
C. flexuosus*C. carvi	Ω_12_	− 625.31	− 2.09	0.0908	− 580.25	− 1.80	0.1311
C. flexuosus*A. calamus	Ω_13_	− 339.26	− 1.13	0.3081	− 739.60	− 2.30	0.0698
C. carvi*A. calamus	Ω_23_	59.63	0.20	0.8498	− 169.20	− 0.53	0.6214
C. flexuosus*C. carvi*A. calamus	Ω_123_	− 4285.57	-2.63	0.0463*	− 4790.40	− 2.74	0.0409*

*Statistically significant at *p* < 0.05.

The statistically significant coefficients for DPPH_IC50_ response are those indicating the effects of individual components (Ω_1_, Ω_2_, and Ω_3_), as well as the ternary interaction term Ω _123_. However, the coefficients of the binary interaction terms (Ω_12_, Ω_13_, and Ω_23_) are nonsignificant (*p* > 0.05) and indicate no impact on DPPH radical. Indeed, after excluding all non-significant coefficients from the assumed models, the mathematical models describing the response as a function of the tested components are expressed by Eq. (3):

3 $${Y}_{DPPH-IC50}=893.10{X}_{1}+207.53{X}_{2}+348.14{X}_{3}-4285.57{X}_{1}{X}_{2}{X}_{3}+\varepsilon$$

Concerning the ABTS_IC50_ response the significant terms were Ω_1_, Ω_3_ and Ω_123_. These outcomes affirm that the antioxidant potential on ABTS radical depends especially on the effect of *C. flexuosus* and *A. calamus* EOs as well as the ternary effect.

Thus, the adopted mathematical model is expressed by Eq. (4):

4 $${Y}_{ABTS-IC50}=1064.19{X}_{1}+200.88{X}_{3}-4790.40{X}_{1}{X}_{2}{X}_{3}+\varepsilon$$

#### Optimization of formulation and desirability study

The optimization operation adopting the experimental design methodology consists of identification of the optimum modulation of the studied components proportions, resulting in the best response values. While the statistically confirmed mathematical models are expected, these optimal outcomes do not necessarily align with those reported in the 12 performed tests; however, they could predict them with high accuracy in the investigated experimental area^55^. In order to gain valuable insights into the best attainable values, we start with the better value when executing the tests^56^. Hence, the best documented results were 185.34 ± 1.07 and 82.25 ± 1.06 µg/mL for DPPH_IC50_ and ABTS_IC50_, respectively. As a result, a setting of the factors enabling the attainment of responses higher than or equal to these readings was accepted.

### Mixture profile

**Figure 4:**
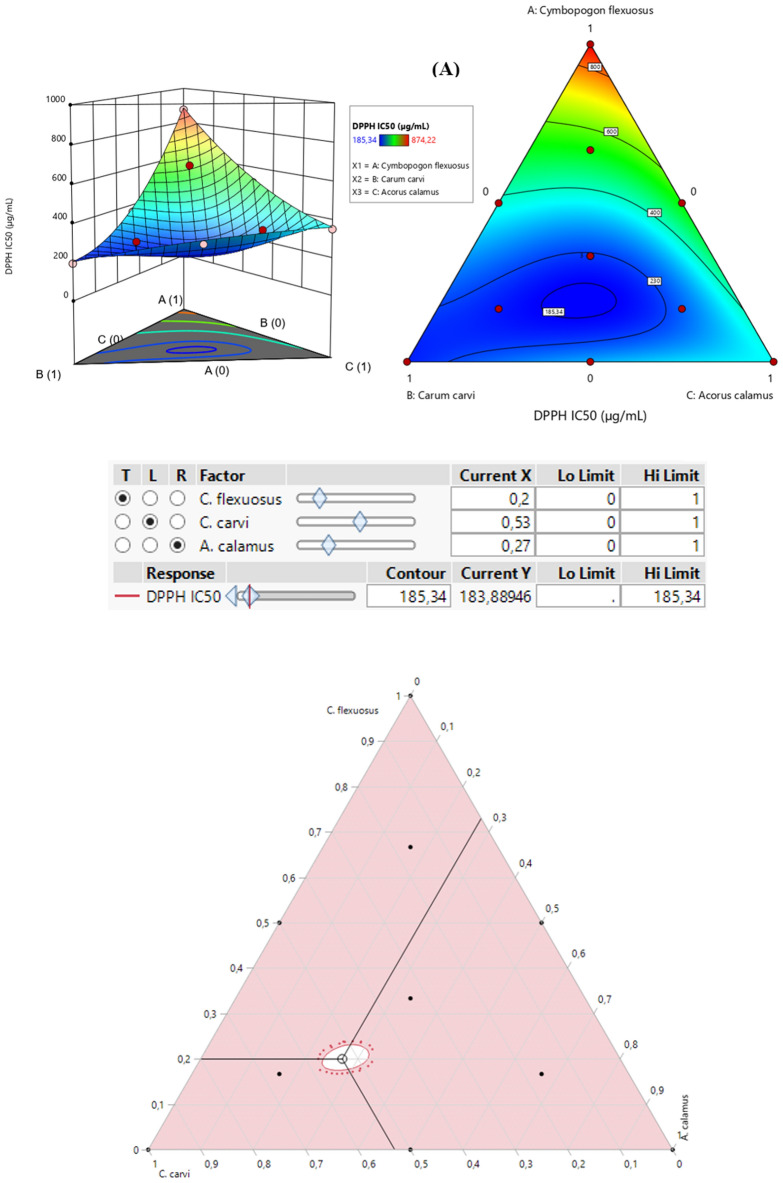

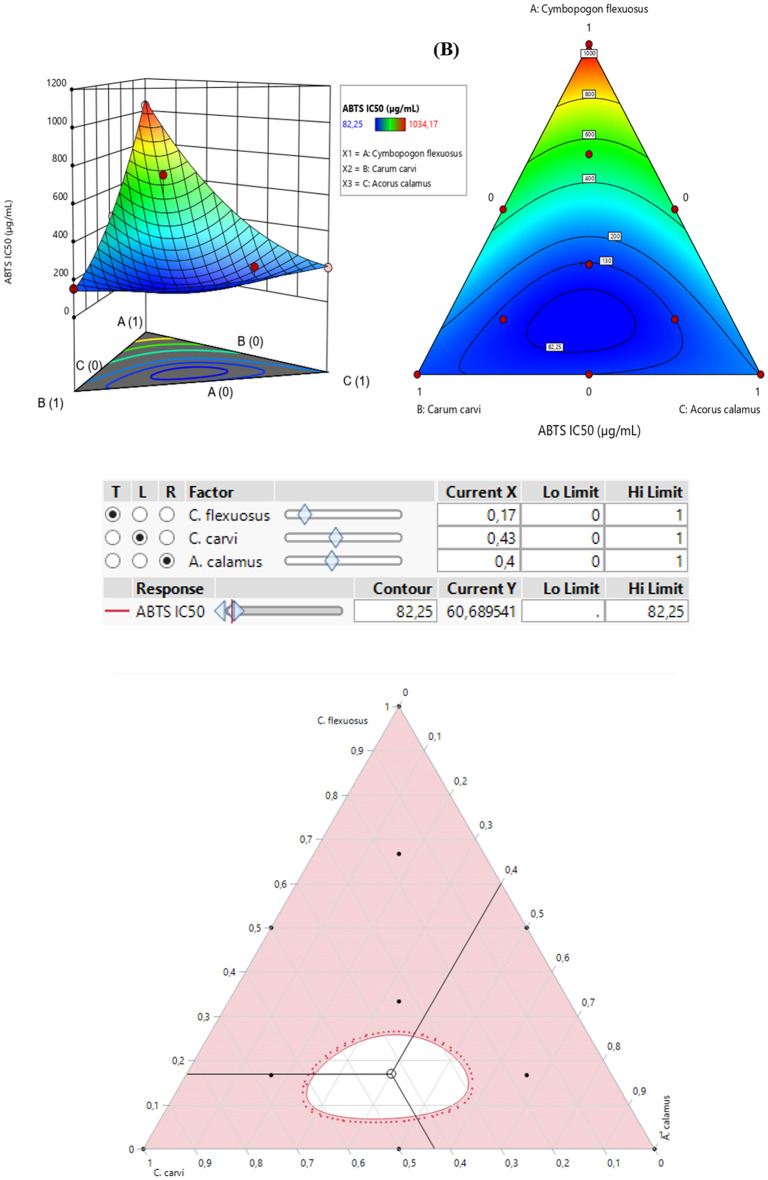
2D and 3D mixture plots of the desired compromise area, resulting in the best value of DPPH_IC50_ (**A**), and ABTS_IC50_ (**B**).

The contour plot and 3D surface (2D and 3D mixture plots, respectively) in Fig. 4 illustrate the optimal combination of the three EO extracts, namely *C. flexuosus*, *C. carvi* and *A. calamus* that maximize both responses (DPPH_IC50_ and ABTS_IC50_). This graph demonstrated the relationship between the responses and the proportions of each antioxidant agent^34,57^. It is produced by the Design-Expert software based on iso-responses curves, which are perfectly suited for examining the ideal conditions to achieve the best response values. The blue color indicate lower IC_50_ values and higher antioxidant potential, whereas yellow to dark red color signifies moderate to higher IC_50_ values.

#### *Optimization of DPPH*_*IC50*_

**Figure 5:**
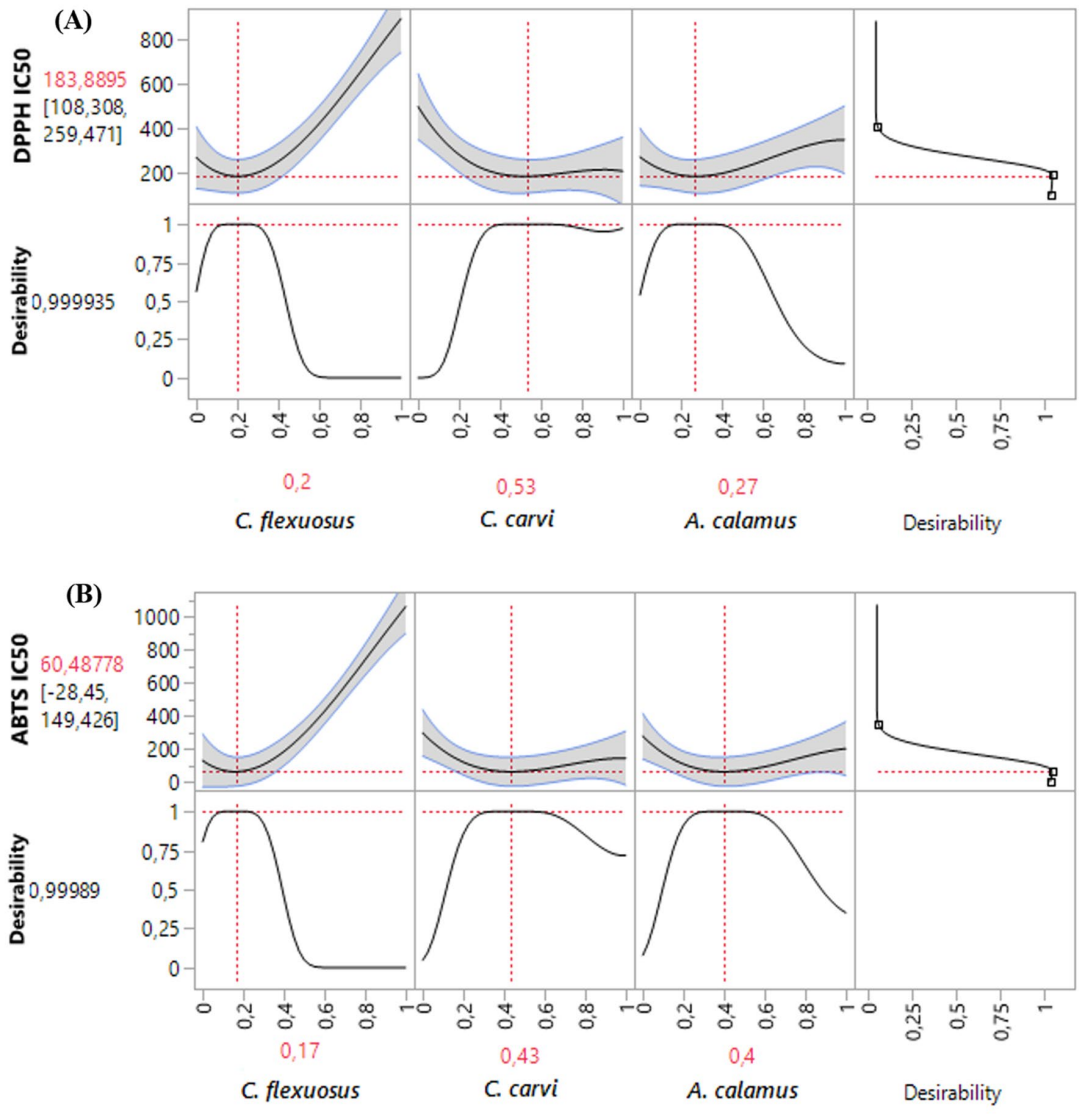
Desirability profile illustrating the precise proportions of *C. flexuosus*, *C. carvi*, and *A. calamus* EOs, leading to the optimum value for DPPH_IC50_ (**A**), and ABTS_IC50_ (**B**), separately.

As depicted in the 2D and 3D mixture plots (Fig. 4A), the optimal compromise area (dark blue zone) corresponding to the best DPPH_-IC50_ value, which equal to 185.34 μg/mL, requires the use of a ternary combination of *C. flexuosus*, *C. carvi* and *A. calamus*. These data are validated by the desirability test (Fig. 5A), in which the optimal value of the DPPH_-IC50_ response that could be reached is equal to 183.88 μg/mL with a compromise percentage of 99.9%. This outcome can be attained by ensuring the ternary mixture with the following proportion: 20% *C. flexuosus*, 53% *C. carvi*, and 27% *A. calamus* EOs. This antioxidant activity is higher than that exhibited by standard antioxidant BHT (IC_50_ = 191.13 ± 0.83 μg/mL).

##### *Optimization of ABTS*_*IC50*_

The contour plot and 3D surface (Fig. 4B) demonstrate that the suggested mixture for ABTS_-IC50_ response also contains the three studied EOs of *C. flexuosus*, *C. carvi*, and *A. calamus*. This combination yields ABTS_-IC50_ response value of approximately 82.25 μg/mL. Moreover, the desirability function in Fig. 5B supports this outcome by demonstrating that a mixture of 17% *C. flexuosus*, 43% *C. carvi*, and 40% *A. calamus*, leads to the best ABTS_-IC50_ value (60.48 μg/mL with 99.98% as the desirability percentage). These findings showed that the optimal combination of the three EOs exhibits strong scavenging ability against ABTS radical as compared to the standard BHT (IC_50_ = 114.07 ± 1.42 μg/mL).

The mixture design methodology has been adopted by several researchers in various disciplines, including the design of EOs mixtures. Baj et al.^58^ optimized the effect of a mixture containing *Ocimum basilicum* L., *Cymbopogon nardus* (L.) Rendle, *Juniperus virginiana* L., and *Thymus vulgaris* L. EOs to achieve high DPPH radical scavenging capacity. In this context, the combined effects of *Petroselinum crispum* (Mill.), *Coriandrum sativum* L., and *Apium graveolens* L. EOs were also optimized using the Simplex Lattice Mixture design^57^.

There has been an increasing interest in recent years to investigate the combined antimicrobial action of EOs by mixtures to achieve the highest antimicrobial effect^27,59–63^. Ouedrhiri et al.^59^ adopted the mixture design methodology to assess the possible synergistic potential of a combined treatment comprising *Myrtus communis* L., *Thymus serpyllum* L., and *Artemisia herba-alba* Asso EOs on *B. subtilis*, *S. aureus* and *E. coli.* Falleh et al.^61^ optimized the amounts of *Lavandula stoechas* L., *Syzygium aromaticum* (L.) Merr. & L.M. Perry, *Myrtus communis* L., and *Cinnamomum zeylanicum* Blume EOs by employing a mixture design technique. As a result, the optimal EOs mixture consisted of 59.4% *C. zeylanicum*, 38.2% *L. stoechas* and 2.4% *S. aromaticum*, which demonstrated synergistic interactions against *E. coli*.

### Simultaneous optimization of all responses

**Figure 6:**
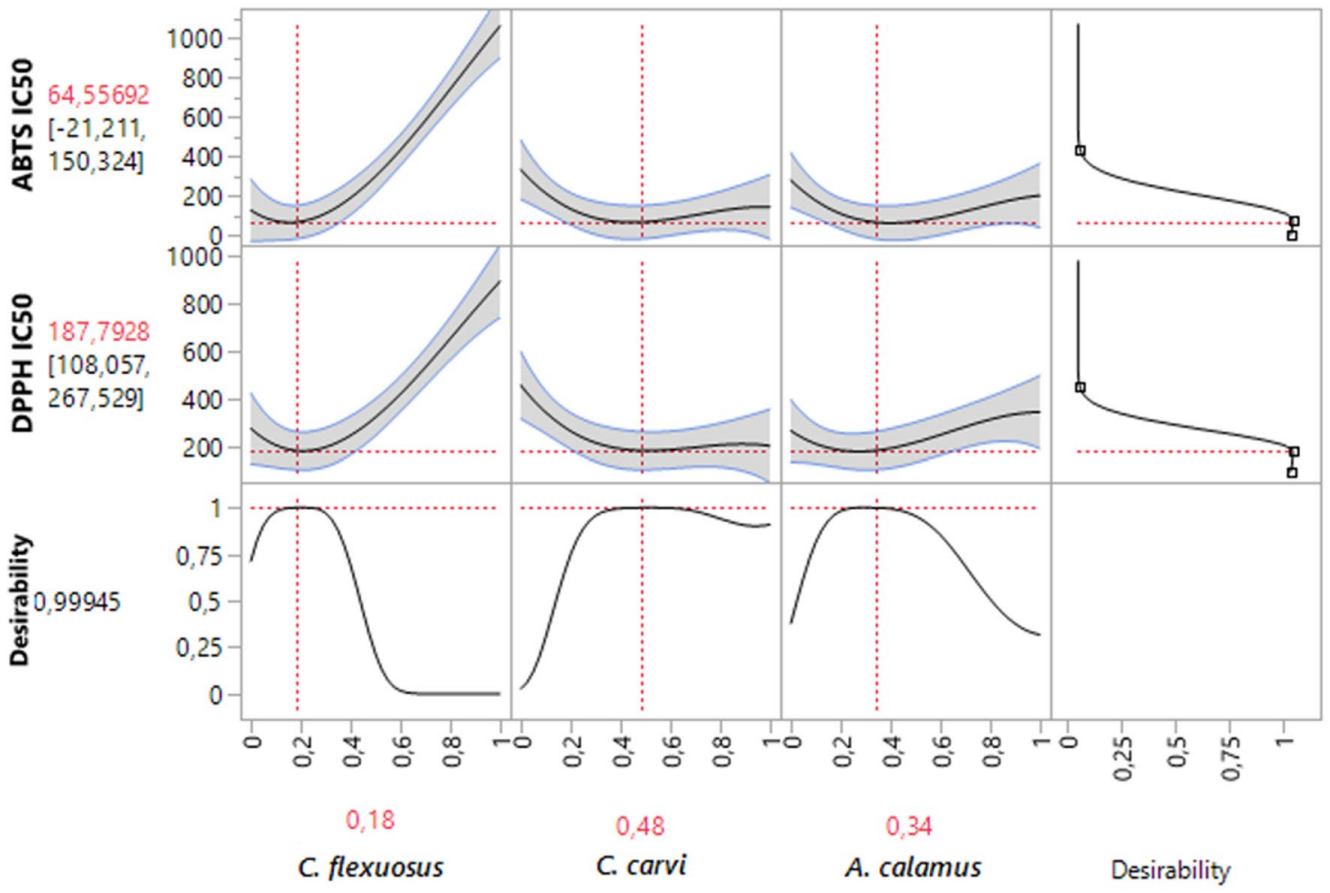
Desirability profile of simultaneous optimization displaying the precise proportions of tested EOs, exhibiting the optimum value for DPPH_IC50_, and ABTS_IC50_.

In addition to its potential to provide accurate results for DPPH_IC50_ and ABTS_IC50_ responses individually, the desirability test also allows identification of the optimal conditions for both investigated responses simultaneously^64^. In our investigation, simultaneous optimization aims to determine the best compromise to enhance the DPPHH_IC50_ and ABTS_IC50_ responses. According to the desirability graph (Fig. 6), the optimization of the two studied responses was possible with a compromise percentage of 99.94% by ensuring the ternary combination with the following proportion: 18% *C. flexuosus*, 48% *C. carvi*, and 34% *A. calamus* EOs. The corresponding optimum response values for this formulation are ff187.79 μg/mL for DPPH_IC50_ and 64.55 μg/mL for ABTS_IC50_.

### Experimental verification of the assumed model

The precision of the cubic models for the two responses, DPPH_IC50_ and ABTS_IC50_ was confirmed by a validation test. The trial involves the comparison of the expected outcomes with the experimental results. The selected test point data represent the EO proportions attained through simultaneous optimization of the two responses. As demonstrated in Table 6, the attained experimental values are strongly correlated with the estimated ones, and no significant difference was noticed between the estimated and experimental responses. The results confirm the accuracy of the choice of the assumed and verified models.

**Table 6:** Expected and observed responses for the test point achieved by the optimal identified mixtures.

Mixture of combination	Proportions of constituents (%)	DPPH_IC50_ (μg/mL)		ABTS_IC50_ (μg/mL)	
		Experimental^a^	Predicted^b^	Experimental^a^	Predicted^b^
*C. flexuosus*	18	186.13 ± 3.02	187.79 ± 5.16	65.98 ± 1.16	64.55 ± 4.11
*C. carvi*	48				
*A. calamus*	34				

^a^The experimental value is the mean of three replicates with standard error.

^b^The predicted value is given with the response's ± SD identified from the model.

## Conclusion

The use of natural formulations has garnered increasing interest. Essential oils possess a wide diversity of chemical constitutions depending on different factors, which may impact simultaneous interactions. Hence, to potentiate their mutual biological effect, statistical tools should be considered. Experimental mixture design may serve as an appropriate method to generate a combination with ideal activities useful for applications in the food or pharmaceutical sector. Herein, we deduce that the optimal antioxidant formulation consists of 20% *C. flexuosus*, 53% *C. carvi*, and 27% *A. calamus* for DPPH_IC50_. Whereas the combination of 17% *C. flexuosus*, 43% *C. carvi*, and 40% *A. calamus* is the best formulation leading to the highest scavenging activity against ABTS radical. This high activity may be related to their bioactive components. Nevertheless, further research on the pharmacokinetic and pharmacodynamics aspects as well as toxicological profile of these formulations is needed to ensure their safety and efficiency.

## Plant collection approval

The authors confirm that no specific approval is needed to collect the studied plants in Morocco for research purposes.

## IUCN policy statement T

The collection of plant material complies with relevant institutional, national, and international guidelines and legislation.

## Data Availability

All data generated or analyzed during this study are included in this published article.
